# Monopolizing Clinical Trial Data: Implications and Trends

**DOI:** 10.1371/journal.pmed.0040002

**Published:** 2007-02-13

**Authors:** Karin Timmermans

## Abstract

Data exclusivity, the granting of exclusive rights over the data required for registration of pharmaceuticals, can jeopardize access to medicines and public health.

The Agreement on Trade-Related Aspects of Intellectual Property Rights (the TRIPS Agreement, Box 1) has to a large extent harmonized standards for intellectual property rights, including patents. For many countries, the TRIPS standards were higher than their previous standards. For example, TRIPS obliges countries to allow patenting of pharmaceuticals and imposes a minimum duration of 20 years for patents. Before TRIPS entered into force, a number of (developing) countries either did not grant patents for medicines, or had a shorter patent term. Since generic medicines can only be marketed in the absence of a patent or after its expiry, the implementation of TRIPS in those countries means it will take longer before generic versions of new medicines can enter the market. The TRIPS Agreement has therefore been criticized for its anticipated detrimental effect on access to medicines, especially in developing countries.

But while much of the debate on TRIPS, intellectual property rights, and access to medicines has focused on patents (Box 2), largely outside the limelight the rather abstract notion of data exclusivity has quietly been introduced and promoted. *Data exclusivity* refers to the granting of exclusive rights over the data required for registration of pharmaceuticals, notably the clinical and preclinical trial data. Data exclusivity, too, can jeopardize access to medicines and negatively affect public health. This article tries to demystify the concept and implications of “data exclusivity,” and to provide an overview of current trends.

## Scrutinizing TRIPS

It has at times been argued that a relatively obscure clause in the TRIPS Agreement—namely its Article 39.3—requires countries to implement data exclusivity [1,2]. However, careful reading of the article does not warrant this conclusion. Article 39.3 essentially demands that undisclosed registration data about new chemical entities be protected against unfair commercial use and against disclosure. Thus, in line with standard regulatory practice, authorities may not publish or share such data—though, importantly, TRIPS does not prevent disclosure when it is necessary to protect the public.

**Box 1: TRIPS and TRIPS-Plus**

The TRIPS Agreement harmonizes standards for various types of intellectual property rights, such as copyrights, patents, and trademarks. TRIPS is an integral part of the WTO Agreements, which create binding obligations among WTO member countries. TRIPS is subject to the WTO's dispute settlement mechanism, which may—as a last resort—allow WTO member countries to apply trade sanctions against a noncompliant country. This is a powerful enforcement mechanism, especially vis-à-vis developing countries, which can usually ill afford to be faced with trade sanctions.

Meanwhile, intellectual property protection that surpasses the standards and requirements of the TRIPS Agreement is often referred to as “TRIPS-plus.” There are many different TRIPS-plus provisions. For example, patent term extensions enable prolongation of the patent term beyond the 20 years required by TRIPS, under certain circumstances. Data exclusivity and “linkage” (see text) are other TRIPS-plus provisions. These TRIPS-plus provisions all delay or hamper generic competition.

Discussions about data exclusivity, however, gravitate around the interpretation of “unfair commercial use” of registration data. Before registering a pharmaceutical product and allowing it on the market, regulatory authorities verify its quality, safety, and efficacy. In the case of a new medicine, safety and efficacy are established via preclinical and clinical trials; hence submission of the trial data is an important prerequisite for registration.

Meanwhile, in order to obtain marketing authorization for their products, generic manufacturers have to submit their own data on quality. In addition, they usually have to demonstrate that their product is chemically and biologically equivalent to the original. When those requirements are satisfied, the regulatory authority will normally assume that the efficacy and safety profiles of both products are the same, and on that basis allow marketing of the generic. Thus, while it could be argued that generic manufacturers indirectly rely on the originator's safety and efficacy data, such manufacturers do not use the originator's data—in fact they do not even have access to them.

The regulatory body relies on the originator's data, but normally does not actually use or revisit them. Moreover, even if the regulatory body would use those data, this would not be commercial use—though such use could, indirectly, have commercial implications. Finally, it does not seem justified to suddenly label longstanding regulatory practices as “unfair.”

**Box 2: Patents, Registration, and Marketing of Medicines**

The pharmaceutical market is highly regulated. Two sets of laws and regulations play a crucial role in shaping this market: the intellectual property laws and the laws and regulations pertaining to drug registration. Intellectual property rights, especially patents, confer negative rights: if a particular medicine is under patent, the patent holder can prevent others from producing or selling (generic versions of) that medicine in the country concerned. But a patent does not give the patent holder the right to put that medicine on the market. In order to be allowed on the market, a medicine has to be registered by the national drug regulatory authority.

Moreover, a patent applies to an invention, not to a medicine per se. Patents can be granted for instance for a new chemical entity, a production process, or a particular formulation. Thus, a single medicine can be covered by more than one patent. Some patents (notably those on the chemical entity) completely block generics. But in other cases it may be possible to produce a generic version without infringing the patent, e.g., a tablet would not infringe a patent that only covers liquid dosage forms.

Recently the independent Commission on Intellectual Property Rights, Innovation and Public Health, established by the World Health Organization, also found that Article 39.3 does not create property rights over registration data, nor does it amount to data exclusivity [3]. This interpretation is further supported by the article's negotiating history [4].

## Exclusivity Examined

Although data exclusivity is not mandated by TRIPS, the European Union (EU), the United States, and a few other countries have chosen to provide for data exclusivity domestically, and are encouraging other countries to follow suit [2,5]. Therefore it is important to be aware of its implications.

Data exclusivity essentially prevents regulatory authorities from relying on data submitted by originator companies in order to register a generic product. By implication, as long as the exclusivity lasts, generic producers would have to submit their own safety and efficacy data. This would oblige them to repeat clinical and preclinical trials—something that takes time and that they usually cannot afford. But more importantly, the repetition of clinical trials raises serious ethical questions, since it would imply withholding medicines that are already known to be effective from some patients (the control group), solely for commercial purposes. It is unlikely that withholding medicines in this way this would pass the scrutiny of ethical review committees, which renders it de facto impossible for generic companies to repeat the clinical trials.

Alternatively, generic manufacturers would have to postpone the launch of their product until the end of the exclusivity period. Thus, data exclusivity can delay generic competition and the ensuing price reductions.

From the perspective of public health and enhancing access to medicines, another troublesome feature of data exclusivity is its potential interference with a compulsory license. A compulsory license is a license, granted by the government (without the agreement of the patent holder) to allow third parties to produce generic versions of a product that is still under patent. Compulsory licensing is an important safeguard-mechanism in TRIPS. Yet data exclusivity could prevent the registration—and hence the actual sale and use—of generics produced under a compulsory license (see Box 3) [6].

The duration of data exclusivity is usually shorter than patent protection; therefore data exclusivity is most relevant when a product has not been patented in a particular country, or when patents can be challenged or circumvented (Box 4).

It is also relevant when a new use or indication is found for an existing medicine whose patent has expired, or is about to expire, since, in order to obtain permission to market a drug for a novel indication, new clinical trial data need to be submitted to the regulatory authority. Registration for a new indication could trigger a new period of exclusivity. Meanwhile, patent laws may not permit the patenting of such a “new indication” (although this is allowed in some jurisdictions). Thus, data exclusivity acquires considerable commercial significance against the backdrop of disappointing levels of discovery and development of new drugs [7–9] and of the struggle by drug companies to extend exclusivity of their top-selling products. According to one commentator: “Drug companies have learned that when they can't create a new drug to treat an existing illness, they can create a new illness to treat with existing drugs” [10]. Data exclusivity, in other words, provides a mechanism that can be used to stave off generic competition.

## Options for Damage Control

Faced with incessant demands, some countries have opted to provide data exclusivity, while trying to mitigate its negative impact on their domestic industries and on access to medicines. They have devised several strategies for damage control.

### Limiting the duration of data exclusivity, and/or specifying that data exclusivity cannot extend beyond the patent term.

The latter strategy was, until recently, explicitly provided for under EU regulations, and was implemented by Greece, Portugal, and Spain [11,12].

**Box 3: Avian Flu and Data Exclusivity in Europe**

In the face of a possible pandemic of avian flu, combined with insufficient stockpiles of the “flu drug” oseltamivir and a global demand that was significantly exceeding the production capacity, questions have been raised in the EU about the role of generic production. The laws of EU member states contain provisions for compulsory licensing, which could be used to allow production of a generic version of a patented medicine. But European legislation does not provide for exceptions to the data exclusivity period following registration of a new medicine. Thus, even if a compulsory license were issued during that period, generic production and marketing would not be allowed, unless the manufacturer conducted its own preclinical tests and clinical trials. Alternatively, the originator would have to agree to the generic competitor's reliance on its data. European officials have stated that they can not waive these requirements, not even in the case of an emergency or outbreak [52].

### Limiting the scope of data exclusivity.

This can be done by specifying explicitly that data exclusivity will only apply to new chemical entities and will not extend to new indications or different formulations of existing medicines. This strategy has been adopted by Egypt and Chile [13,14].

### Imposing quick registration of a medicine.

Chile has drafted regulations specifying that failure to register a new medicine in Chile within one year after obtaining the first global marketing authorization will disqualify it for data exclusivity [14].

### Creating procedures for “compulsory licensing” of the data that fall under the exclusive rights.

This strategy draws on US practices in case of mergers [6] and on the examples of Costa Rica and Brazil [13].

### Enabling health authorities to waive data exclusivity when it is deemed in the interest of public health or of specific patients to do so.

This strategy is analogous to the registration waivers or “compassionate use” provisions that often figure in national rules on drug registration—Colombia reportedly takes this line [13]. Waiving data exclusivity is also the approach followed by the EU in the case that a compulsory license is issued to allow the production of generic pharmaceuticals for export to countries that lack production capacity [15].

In other cases, regulators do not rely on the originator's confidential safety and efficacy data when registering a generic medicine. Instead, they rely on published data or on foreign registration of the medicine concerned —Argentina for instance has been said to use the latter approach [1]. In fact, referring to or relying on foreign registration is a longstanding, recommended practice, especially for regulatory authorities with limited (human) resources [16–20].

Finally, there have been proposals to allow use of clinical trial data by generic competitors on a cost-sharing basis. Cost-sharing would prevent the creation of new monopoly rights, but instead enable competition in return for a fair, and probably modest, compensation to the originator of the data [13].

## Bilateral Agreements: Preemptive Strikes?

Meanwhile, on the trade front, countries are increasingly turning to bilateral and regional free trade negotiations. At the instigation of their well-established pharmaceutical industry [21–23], some developed countries are using these negotiations to obtain protection for intellectual property that goes significantly beyond the TRIPS standards. Data exclusivity figures prominently among those “TRIPS-plus” requirements (Box 1).

A comparison of bilateral free trade agreements (FTAs) that have been concluded in recent years between the US and an array of other countries demonstrates a worrisome trend: the requirements for data exclusivity are progressively getting tighter (Table 1). FTAs also increasingly preclude the use of the strategies for damage control discussed above.

**Table 1:**
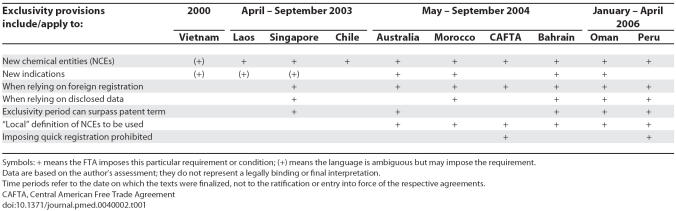
Overview of Data Exclusivity Provisions in Recent US FTAs

In line with the tendency to seek ever more detailed and stringent data exclusivity concessions in FTAs, shown in Table 1, Thailand reportedly is facing extensive demands in this area during its bilateral trade negotiations with the US [24–26]. Moreover, Thailand risks being faced with similar demands during concurrent negotiations with the European Free Trade Association (EFTA) [27]. This risk is not imaginary, since the EFTA has already concluded several other free trade agreements that contain “TRIPS-plus” provisions, including data exclusivity [28–30].

**Box 4: Affecting Access to Antiretrovirals**

In China, one of the key first-line antiretrovirals for treatment of HIV/AIDS is protected by process patents, which can be circumvented. There is no molecular patent that would completely block generic production, and Chinese manufacturers reportedly are producing the active pharmaceutical ingredient (or raw material) for export. But because of “administrative protection” (the Chinese equivalent of data exclusivity), these companies are not allowed to market the final product (tablets) to patients inside China that need them [53].

Meanwhile in Guatemala, where most antiretrovirals are not under patent, Médecins Sans Frontières is treating AIDS patients mostly with generic medicines. Their considerably lower prices (5%–50% of the price of originator products) have made it possible to expand access to first line treatment. However, Médecins Sans Frontières has expressed concern that recently enacted data exclusivity provisions will preclude the use of generic versions of newer antiretrovirals such as atazanavir, and could thus render second-line treatment unaffordable [54,55].

Perhaps even more disturbing are suspicions that the EU may be attempting to include requirements for European-style data exclusivity in its Economic Partnership Agreements [31,32]; the EU already expects data exclusivity from new and aspirant member states [33,34]. EU-style data exclusivity lasts longer and hence could impede access to medicines even more seriously than US-type provisions (Box 5).

## Blurring the Boundaries

The boundaries between the registration system and the intellectual property system are further blurred by requirements that the regulatory authority should withhold registration of generic versions of patented drugs. This is often referred to as “linkage.” Currently generic companies are free to make their own assessment as to whether a patent would stand up to legal scrutiny; when they consider a patent weak, generic manufacturers may decide to enter the market regardless.

“Linkage” renders the regulatory authority de facto responsible for enforcing pharmaceutical patents. When implementing it, regulators—having neither the expertise, the resources, nor the mandate to assess the validity of a patent—will probably enforce any and all patents. This is problematic; in the US, generic companies have regularly prevailed in pharmaceutical patent infringement cases [35], meaning that in those cases the patent was either not infringed or not valid.

Thus, making registration conditional upon the absence of a patent will create additional barriers for generic manufacturers. It will also redouble the incentives for “evergreening”: the practice of filing additional and at times frivolous patents on minor improvements, or even simply on particular features of existing medicines, in an effort to keep generic competition at bay. Unfortunately—though perhaps not surprisingly—virtually all recent bilateral FTAs concluded by the US contain clauses mandating “linkage” between registration and patent status.

**Box 5: Data Exclusivity in the EU Versus the US**

In the EU, data exclusivity for a new medicinal product lasts for eight years and is followed by two years of market exclusivity. During the latter period, regulatory authorities can accept and evaluate the registration dossier of a generic version of the same product, though marketing can only commence at the end of the entire ten year exclusivity period. These ten years can be extended by one year if, during the first eight years of the exclusivity period, the product has been registered for one or more new indications for which it is believed to be of “significant clinical benefit” [56]. Meanwhile, in the US, data exclusivity lasts five years for products with a new active ingredient, and three years for a new indication of a known product [57]. However, in the US, multiple extensions appear to be possible, while EU regulations allow only a single extension.

## Accidents on Accession?

As if the above is not troubling enough, another worrisome trend has started to emerge: “TRIPS-plus” requirements are being imposed during World Trade Organization (WTO) accession negotiations. While initially appearing to be an incident unique to the accession of China [36], more recently, acceptance of tightly worded provisions on data exclusivity seems to have become a rather routine precondition for aspiring WTO members.

The first indication that this precondition was becoming more common was the reference to data exclusivity during Cambodia's accession [37]. The alarm bells that sounded when this fact became known [38–40] apparently were heard, and during the formal acceptance of Cambodia's WTO membership, reference was made to the Doha Declaration on the TRIPS Agreement and Public Health [41], by virtue of which Cambodia would be able to defer the implementation of data protection.

But all this was quickly forgotten thereafter; similar “TRIPS-plus” commitments have surfaced during the recent accession of Tonga [42]. Data exclusivity has also been raised during the accessions of Saudi Arabia and Vietnam [43,44], and there are reports and fears that “TRIPS-plus” concessions are being asked of Russia [45], which is actively negotiating its way into the WTO.

Moreover, there appears to be little mercy for the small or the weak: the accession documents of Cambodia (a least-developed country) and Tonga (a small pacific island nation) not only contain obligations with regard to data exclusivity, but also explicitly impose “linkage”—a feature that thus far is unique to the accession of small or least-developed countries.

## Stemming the Tide?

Table 1 shows how FTAs are increasingly used to micromanage other countries' domestic policies. WTO accession negotiations risk becoming an extension of this strategy. These trends beg the question of what competition in the pharmaceutical sector will look like in the future, and create serious concerns about the prospects for access to medicines, especially in developing countries.

But maybe all is not yet lost. The Southern African Customs Union has not yet caved in to “TRIPS-plus” demands in bilateral negotiations with both the US and EFTA [46–51]. Meanwhile, the emerging trend of making use of WTO accession negotiations to advance the ”TRIPS-plus” agenda—which goes against the spirit of the Doha Declaration on TRIPS and Public Health—can probably still be nipped in the bud, if current WTO members recognize what is happening and take a common stance against the few demanders.

But more countries should resist demands that monopolize the use of clinical trial data and blur the boundaries between the intellectual property regime and regulatory requirements for pharmaceuticals. And the health sector should pay more attention to these developments outside its immediate purview, wake up to the far-reaching implications of these developments, and voice its concerns more widely and more effectively. Failing that, the battle for access to medicines will be lost on these new and little-known fronts.

## Abbreviations

EFTA: European Free Trade Association
EU: European Union
FTA: free trade agreement
TRIPS: Trade-Related Aspects of Intellectual Property Rights
WTO: World Trade Organization
